# Supplementary material for: Phase transitions in 2D multistable mechanical metamaterials via collisions of soliton-like pulses
Source: Nat Commun. 2024 Jan 6;15:333. doi: 10.1038/s41467-023-44293-w (PMC10771479; doi:10.1038/s41467-023-44293-w)
Supplement: Supplementary file 1 — Supplementary Information [file 41467_2023_44293_MOESM1_ESM.pdf]

Supplementary Information for
Phase transitions in 2D multistable mechanical metamaterials via collisions of soliton-like pulses

Weijian Jiao,[1,2] Hang Shu,[1] Vincent Tournat,[3] Hiromi Yasuda,[1,4] and Jordan R. Raney[1,*]

[1]Department of Mechanical Engineering and Applied Mechanics,
University of Pennsylvania, Philadelphia, Pennsylvania 19104, USA
[2]School of Aerospace Engineering and Applied Mechanics, Tongji University, Shanghai 200092, China
[3]Laboratoire d'Acoustique de l'Université du Mans (LAUM), UMR 6613,
Institut d'Acoustique - Graduate School (IA-GS), CNRS, Le Mans Université, France
[4]Aviation Technology Directorate, Japan Aerospace Exploration Agency, Mitaka, Tokyo 1810015, Japan

Supplementary Note 1: Equations of motion

Based on the discrete model introduced in the main text, the Hamiltonian of a 2D rotating-squares system can be written as

$$\begin{aligned}
H = &\frac{1}{2}\sum_{n,m}\left(M\dot{u}_{n,m}^2 + M\dot{v}_{n,m}^2 + J\dot{\theta}_{n,m}^2\right) + \sum_{n,m}\Big[V_{Morse}(\Delta\theta_{n-1,m}) + V_{Morse}(\Delta\theta_{n,m-1})\Big] \\
&+ \frac{1}{2}K_\theta\sum_{n,m}\Big[(\theta_{n,m}+\theta_{n-1,m}+2\theta_0-2\theta_{Lin})^2 + (\theta_{n,m}+\theta_{n,m-1}+2\theta_0-2\theta_{Lin})^2\Big] \\
&+ \frac{1}{2}K_l\sum_{n,m}\Big[u_{n,m}-u_{n-1,m}-L\cos(\theta_{n,m}+\theta_0)-L\cos(\theta_{n-1,m}+\theta_0)+2L\cos\theta_0\Big]^2 \\
&+ \frac{1}{2}K_l\sum_{n,m}\Big[v_{n,m}-v_{n,m-1}-L\cos(\theta_{n,m}+\theta_0)-L\cos(\theta_{n,m-1}+\theta_0)+2L\cos\theta_0\Big]^2 \\
&+ \frac{1}{2}K_s\sum_{n,m}\Big[u_{n,m}-u_{n,m-1}-(-1)^{n+m}L\sin(\theta_{n,m}+\theta_0)-(-1)^{n+m-1}L\sin(\theta_{n,m-1}+\theta_0)\Big]^2 \\
&+ \frac{1}{2}K_s\sum_{n,m}\Big[v_{n,m}-v_{n-1,m}+(-1)^{n+m}L\sin(\theta_{n,m}+\theta_0)+(-1)^{n+m-1}L\sin(\theta_{n-1,m}+\theta_0)\Big]^2,
\end{aligned} \tag{1}$$

where $\Delta\theta_{n\pm1,m\pm1} = \theta_{n,m}+\theta_{n\pm1,m\pm1}+2(\theta_0-\theta_{Lin})$ and $L = \frac{a}{2\cos\theta_0}$ is half of the diagonal length of the square. Then, Hamilton's equations read

$$M\ddot{u}_{n,m} = -\frac{\partial H}{\partial u_{n,m}}, \tag{2}$$

$$M\ddot{v}_{n,m} = -\frac{\partial H}{\partial v_{n,m}}, \tag{3}$$

$$J\ddot{\theta}_{n,m} = -\frac{\partial H}{\partial \theta_{n,m}}. \tag{4}$$

Substituting Eq. 1 in Eq. 4, the equations of motion (EOMs) for the square at site (n, m) can be derived as

$$\begin{aligned}
M\frac{\partial^2 u_{n,m}}{\partial t^2} = &K_l\,(u_{n-1,m}+u_{n+1,m}-2u_{n,m}) + K_s(u_{n,m-1}+u_{n,m+1}-2u_{n,m}) \\
&+ K_l\frac{a}{2\cos\theta_0}\Big[\cos(\theta_{n-1,m}+\theta_0)-\cos(\theta_{n+1,m}+\theta_0)\Big] \\
&+ (-1)^{n+m}K_s\frac{a}{2\cos\theta_0}\Big[\sin(\theta_{n,m+1}+\theta_0)-\sin(\theta_{n,m-1}+\theta_0)\Big],
\end{aligned} \tag{5}$$

*Electronic address: raney@seas.upenn.edu

$$
\begin{aligned}
M\frac{\partial^2 v_{n,m}}{\partial t^2} &= K_s(v_{n-1,m}+v_{n+1,m}-2v_{n,m})+K_l(v_{n,m-1}+v_{n,m+1}-2v_{n,m}) \\
&+K_l\frac{a}{2\cos\theta_0}\Big[\cos(\theta_{n,m-1}+\theta_0)-\cos(\theta_{n,m+1}+\theta_0)\Big] \\
&+(-1)^{n+m}K_s\frac{a}{2\cos\theta_0}\Big[-\sin(\theta_{n+1,m}+\theta_0)+\sin(\theta_{n-1,m}+\theta_0)\Big],
\end{aligned}
\tag{6}
$$

$$
\begin{aligned}
J\frac{\partial^2 \theta_{n,m}}{\partial t^2} &= -K_\theta(\theta_{n-1,m}+\theta_{n+1,m}+\theta_{n,m+1}+\theta_{n,m-1}+4\theta_{n,m}+8\theta_0-8\theta_{Lin}) \\
&-K_l\frac{a}{2\cos\theta_0}\sin(\theta_{n,m}+\theta_0)\,(u_{n+1,m}+v_{n,m+1}-u_{n-1,m}-v_{n,m-1}) \\
&-K_l\frac{a^2}{4\cos^2\theta_0}\sin(\theta_{n,m}+\theta_0)\Big[-\cos(\theta_{n+1,m}+\theta_0)-\cos(\theta_{n-1,m}+\theta_0) \\
&-\cos(\theta_{n,m+1}+\theta_0)-\cos(\theta_{n,m-1}+\theta_0)-4\cos(\theta_{n,m}+\theta_0)+8\cos\theta_0\Big] \\
&+(-1)^{n+m}K_s\frac{a}{2\cos\theta_0}\cos(\theta_{n,m}+\theta_0)\,(u_{n,m+1}-u_{n,m-1}+v_{n-1,m}-v_{n+1,m}) \\
&+K_s\frac{a^2}{4\cos^2\theta_0}\cos(\theta_{n,m}+\theta_0)\Big[\sin(\theta_{n+1,m}+\theta_0)+\sin(\theta_{n,m+1}+\theta_0) \\
&-4\sin(\theta_{n,m}+\theta_0)+\sin(\theta_{n-1,m}+\theta_0)+\sin(\theta_{n,m-1}+\theta_0)\Big] \\
&-T_{Morse}(\Delta\theta_{n+1,m})-T_{Morse}(\Delta\theta_{n-1,m})-T_{Morse}(\Delta\theta_{n,m+1})-T_{Morse}(\Delta\theta_{n,m-1}),
\end{aligned}
\tag{7}
$$

where

$$
\begin{aligned}
T_{Morse}(\Delta\theta) &= 2\alpha A\left[e^{2\alpha(\Delta\theta+2\theta_0-2\theta_{Morse})}-e^{\alpha(\Delta\theta+2\theta_0-2\theta_{Morse})}\right] \\
&-2\alpha A\left[e^{-2\alpha(\Delta\theta+2\theta_0+2\theta_{Morse})}-e^{-\alpha(\Delta\theta+2\theta_0+2\theta_{Morse})}\right],
\end{aligned}
\tag{8}
$$

Note, we define the positive direction of rotation with alternating sign for neighboring squares.

By introducing $K_1 = K_s/K_l$, $K_2 = k_\theta/(K_l a^2)$, $T = t\sqrt{K_l/M}$, $\beta = a\sqrt{M/J}$, $\bar{A} = A/(K_l a^2)$, $U = u/a$, $V = v/a$, we can obtain the following dimensionless EOMs

$$
\begin{aligned}
\frac{\partial^2 U_{n,m}}{\partial T^2} &= (U_{n-1,m}+U_{n+1,m}-2U_{n,m})+K_1(U_{n,m-1}+U_{n,m+1}-2U_{n,m}) \\
&+\frac{1}{2\cos\theta_0}\Big[\cos(\theta_{n-1,m}+\theta_0)-\cos(\theta_{n+1,m}+\theta_0)\Big] \\
&+(-1)^{n+m}\frac{K_1}{2\cos\theta_0}\Big[\sin(\theta_{n,m+1}+\theta_0)-\sin(\theta_{n,m-1}+\theta_0)\Big],
\end{aligned}
\tag{9}
$$

$$
\begin{aligned}
\frac{\partial^2 V_{n,m}}{\partial T^2} &= K_1(V_{n-1,m}+V_{n+1,m}-2V_{n,m})+(V_{n,m-1}+V_{n,m+1}-2V_{n,m}) \\
&+\frac{1}{2\cos\theta_0}\Big[\cos(\theta_{n,m-1}+\theta_0)-\cos(\theta_{n,m+1}+\theta_0)\Big] \\
&+(-1)^{n+m}\frac{K_1}{2\cos\theta_0}\Big[-\sin(\theta_{n+1,m}+\theta_0)+\sin(\theta_{n-1,m}+\theta_0)\Big],
\end{aligned}
\tag{10}
$$

$$
\begin{aligned}
\frac{1}{\beta^2}\frac{\partial^2\theta_{n,m}}{\partial T^2} &= -K_2(\theta_{n-1,m}+\theta_{n+1,m}+\theta_{n,m+1}+\theta_{n,m-1}+4\theta_{n,m}+8\theta_0-8\theta_{Lin}) \\
&- \frac{\sin(\theta_{n,m}+\theta_0)}{4\cos^2\theta_0}\Big[2\cos\theta_0(U_{n+1,m}+V_{n,m+1}-U_{n-1,m}-V_{n,m-1}) \\
&- \cos(\theta_{n+1,m}+\theta_0)-\cos(\theta_{n-1,m}+\theta_0)-\cos(\theta_{n,m+1}+\theta_0) \\
&- \cos(\theta_{n,m-1}+\theta_0)-4\cos(\theta_{n,m}+\theta_0)+8\cos\theta_0\Big] \\
&+ \frac{(-1)^{n+m}K_1}{4\cos^2\theta_0}\cos(\theta_{n,m}+\theta_0)\Big[2\cos\theta_0(U_{n,m+1}-U_{n,m-1}+V_{n-1,m}-V_{n+1,m})\Big] \\
&+ \frac{K_1}{4\cos^2\theta_0}\cos(\theta_{n,m}+\theta_0)\Big[\sin(\theta_{n+1,m}+\theta_0)+\sin(\theta_{n,m+1}+\theta_0) \\
&- 4\sin(\theta_{n,m}+\theta_0)+\sin(\theta_{n-1,m}+\theta_0)+\sin(\theta_{n,m-1}+\theta_0)\Big] \\
&- \bar{T}_{Morse}(\Delta\theta_{n+1,m})-\bar{T}_{Morse}(\Delta\theta_{n-1,m})-\bar{T}_{Morse}(\Delta\theta_{n,m+1})-\bar{T}_{Morse}(\Delta\theta_{n,m-1}),
\end{aligned} \tag{11}
$$

where $\bar{T}_{Morse} = T_{Morse}/(K_l a^2)$. The dimensionless EOMs of the system can be obtained by considering Eqs. 9-11 for all squares. Then, full-scale simulations can be conducted by numerically solving the system's EOMs using the fourth order Runge-Kutta method (via the Matlab function *ode45*). Based on preliminary numerical results, we observe that, after a transition wave is initiated, squares in the new phase can undergo large oscillations due to the energy release from the initial Phase C to the new Phase R. To account for the disspision observed in the experiments, we include damping in the simulations by introducing the following simple viscous damping terms in the EOMs: $c_u = \lambda_u \frac{\partial U}{\partial T}$, $c_v = \lambda_v \frac{\partial V}{\partial T}$, and $c_\theta = \lambda_\theta \frac{\partial \theta}{\partial T}$, in which λ_u and λ_v are damping coefficients for translational motion in the x and y directions, respectively, and λ_θ is the damping coefficient for rotational motion. For simulations of the nucleation via quasitatic loading, $\lambda_u = \lambda_v = 4.824\times10^{-4}$ and $\lambda_\theta = 0.173$. For simulations of the nucleation via collisions of soliton-like pulses, $\lambda_u = \lambda_v = 1.508\times10^{-4}$ and $\lambda_\theta = 0.057$. Damping is added only after the new phase is formed in the simulations.

Supplementary Note 2: Continuum model

Previous studies [1,2] have shown that analytical solutions of solitons are available in similar but monostable systems of squares without magnets. We expect that soliton can propagate in our system as long as it stays within the energy well around the initial equilibrium θ_0. To derive analytical solutions for solitons, we approximate the total normalized torsional potential, including both elastic torsional potential and Morse potential, by a forth order polynomial:

$$
V_t \approx C_0 + \frac{C_2}{2}\Delta\theta^2 + \frac{C_3}{3}\Delta\theta^3 + \frac{C_4}{4}\Delta\theta^4, \tag{12}
$$

in which the linear torsional potential is incorporated in the second-order term.

Following the procedures presented in [2], we introduce three continuous functions $U(X,Y,T)$, $V(X,Y,T)$ and $\theta(X,Y,T)$, which interpolate the discrete variables $U_{n,m}$, $V_{n,m}$ and $\theta_{n,m}$ as

$$
\begin{aligned}
U\,(X=n,\,Y=m,\,T) &= U_{n,m}, \\
V\,(X=n,\,Y=m,\,T) &= V_{n,m}, \\
\theta\,(X=n,\,Y=m,\,T) &= \theta_{n,m},
\end{aligned} \tag{13}
$$

Assuming that the width of the propagating wave is much larger than the unit cell, the displacements U and V and the rotation θ of the $[n,\,m-1]$-th, $[n,\,m+1]$-th, $[n-1,\,m]$-th, and $[n+1,\,m]$-th units can then be expressed using Taylor expansion as

$$
\begin{aligned}
F_{n\pm1,m} &\approx F|_{n,m} \pm \left.\frac{\partial F}{\partial X}\right|_{n,m} + \frac{1}{2}\left.\frac{\partial^2 F}{\partial X^2}\right|_{n,m} \pm \frac{1}{6}\left.\frac{\partial^3 F}{\partial X^3}\right|_{n,m}, \\
F_{n,m\pm1} &\approx F|_{n,m} \pm \left.\frac{\partial F}{\partial Y}\right|_{n,m} + \frac{1}{2}\left.\frac{\partial^2 F}{\partial Y^2}\right|_{n,m} \pm \frac{1}{6}\left.\frac{\partial^3 F}{\partial Y^3}\right|_{n,m},
\end{aligned} \tag{14}
$$

where $F \in U, V, \theta$. Substitution of Eq. (12) and Eq. (14) into Eqs. (9)-(11) yields

$$
\frac{\partial^2 U}{\partial T^2} = \frac{\partial^2 U}{\partial X^2} + K_1\frac{\partial^2 U}{\partial Y^2} - \frac{1}{2\cos\theta_0}\left[2\frac{\partial\cos(\theta+\theta_0)}{\partial X} + \frac{1}{3}\frac{\partial^3\cos(\theta+\theta_0)}{\partial X^3}\right], \tag{15}
$$

$$\frac{\partial^2 V}{\partial T^2} = K_1 \frac{\partial^2 V}{\partial X^2} + \frac{\partial^2 V}{\partial Y^2} - \frac{1}{2\cos\theta_0}\left[2\frac{\partial \cos(\theta+\theta_0)}{\partial Y} + \frac{1}{3}\frac{\partial^3 \cos(\theta+\theta_0)}{\partial Y^3}\right], \tag{16}$$

$$\begin{aligned}\frac{1}{\beta^2}\frac{\partial^2\theta}{\partial T^2} &= -C_2\left(\frac{\partial^2\theta}{\partial X^2} + \frac{\partial^2\theta}{\partial Y^2} + 8\theta\right) - 16C_3\theta^2 - 32C_4\theta^3 \\ &- \frac{1}{4\cos^2\theta_0}\sin(\theta+\theta_0)\left[4\cos\theta_0\left(\frac{\partial U}{\partial X} + \frac{\partial V}{\partial Y}\right) - \frac{\partial^2\cos(\theta+\theta_0)}{\partial X^2} - \frac{\partial^2\cos(\theta+\theta_0)}{\partial Y^2} - 8\cos(\theta+\theta_0) + 8\cos\theta_0\right] \\ &+ K_1\frac{1}{4\cos^2\theta_0}\cos(\theta+\theta_0)\left[\frac{\partial^2\sin(\theta+\theta_0)}{\partial X^2} + \frac{\partial^2\sin(\theta+\theta_0)}{\partial Y^2}\right].\end{aligned} \tag{17}$$

It is important to note that in deriving the above equations we disregarded all the terms that switch sign between each neighboring unit (i.e., the terms with coefficient $(-1)^{n+m}$). Moreover, if we assume that $\theta \ll 1$, $\sin(\theta_0+\theta)$ and $\cos(\theta_0+\theta)$ can be approximated as

$$\begin{aligned}\sin(\theta_0+\theta) &\approx \sin\theta_0 + \theta\ \cos\theta_0 - \theta^2\frac{\sin\theta_0}{2} - \theta^3\frac{\cos\theta_0}{6}, \\ \cos(\theta_0+\theta) &\approx \cos\theta_0 - \theta\ \sin\theta_0 - \theta^2\frac{\cos\theta_0}{2} + \theta^3\frac{\sin\theta_0}{6}.\end{aligned} \tag{18}$$

Substituting Eq. (18) into Eqs. (15)-(17) and retaining the nonlinear terms up to the third order yields

$$\frac{\partial^2 U}{\partial T^2} = \frac{\partial^2 U}{\partial X^2} + K_1\frac{\partial^2 U}{\partial Y^2} + \left(\tan\theta_0 + \theta - \frac{1}{2}\tan\theta_0\theta^2\right)\frac{\partial\theta}{\partial X} + \frac{\tan\theta_0}{6}\frac{\partial^3\theta}{\partial X^3}, \tag{19}$$

$$\frac{\partial^2 V}{\partial T^2} = K_1\frac{\partial^2 V}{\partial X^2} + \frac{\partial^2 V}{\partial Y^2} + \left(\tan\theta_0 + \theta - \frac{1}{2}\tan\theta_0\theta^2\right)\frac{\partial\theta}{\partial Y} + \frac{\tan\theta_0}{6}\frac{\partial^3\theta}{\partial Y^3}, \tag{20}$$

$$\begin{aligned}\frac{1}{\beta^2}\frac{\partial^2\theta}{\partial T^2} &= -\left(8C_2 + 2\tan^2\theta_0\right)\theta - \left(16C_3 + 3\tan\theta_0\right)\theta^2 - \left(32C_4 + 1 - \frac{4\tan^2\theta_0}{3}\right)\theta^3 \\ &+ \frac{K_1 - \tan^2\theta_0 - 4C_2}{4}\left(\frac{\partial^2\theta}{\partial X^2} + \frac{\partial^2\theta}{\partial Y^2}\right) - \left(\tan\theta_0 + \theta - \frac{1}{2}\tan\theta_0\theta^2\right)\left(\frac{\partial U}{\partial X} + \frac{\partial V}{\partial Y}\right).\end{aligned} \tag{21}$$

We now consider a planar wave solution with the traveling coordinate $\xi = X\cos\phi + Y\sin\phi - cT$, where c is the normalized wave speed, and ϕ is the angle between the propagation direction and the x axis. It follows that Eqs. (19)-(21) can be rewritten as

$$\left(c^2 - \cos^2\phi - K_1\sin^2\phi\right)\frac{\partial^2 U}{\partial\xi^2} = \left(\tan\theta_0 + \theta - \frac{1}{2}\tan\theta_0\theta^2\right)\cos\phi\frac{\partial\theta}{\partial\xi} + \frac{\tan\theta_0\cos^3\phi}{6}\frac{\partial^3\theta}{\partial\xi^3}, \tag{22}$$

$$\left(c^2 - K_1\cos^2\phi - \sin^2\phi\right)\frac{\partial^2 V}{\partial\xi^2} = \left(\tan\theta_0 + \theta - \frac{1}{2}\tan\theta_0\theta^2\right)\sin\phi\frac{\partial\theta}{\partial\xi} + \frac{\tan\theta_0\sin^3\phi}{6}\frac{\partial^3\theta}{\partial\xi^3}, \tag{23}$$

$$\begin{aligned}\left(\frac{c^2}{\beta^2} - \frac{K_1 - \tan^2\theta_0 - 4C_2}{4}\right)\frac{\partial^2\theta}{\partial\xi^2} &= -\left(8C_2 + 2\tan^2\theta_0\right)\theta - \left(16C_3 + 3\tan\theta_0\right)\theta^2 - \left(32C_4 + 1 - \frac{4\tan^2\theta_0}{3}\right)\theta^3 \\ &- \left(\tan\theta_0 + \theta - \frac{1}{2}\tan\theta_0\theta^2\right)\left(\cos\phi\frac{\partial U}{\partial\xi} + \sin\phi\frac{\partial V}{\partial\xi}\right).\end{aligned} \tag{24}$$

Integrating Eqs. (22) and (27) with respect to ξ yields

$$\frac{\partial U}{\partial\xi} = \frac{\cos\phi\left(\tan\theta_0\theta + \theta^2/2 - \tan\theta_0\theta^3/6\right)}{c^2 - \cos^2\phi - K_1\sin^2\phi} + \frac{\tan\theta_0\cos^3\phi}{6c^2 - 6\cos^2\phi - 6K_1\sin^2\phi}\frac{\partial^2\theta}{\partial\xi^2} + B_1, \tag{25}$$

$$\frac{\partial V}{\partial\xi} = \frac{\sin\phi\left(\tan\theta_0\theta + \theta^2/2 - \tan\theta_0\theta^3/6\right)}{c^2 - K_1\cos^2\phi - \sin^2\phi} + \frac{\tan\theta_0\sin^3\phi}{6c^2 - 6K_1\cos^2\phi - 6\sin^2\phi}\frac{\partial^2\theta}{\partial\xi^2} + B_2, \tag{26}$$

where B_1 and B_2 are integration constants. Since we are interested in a soliton solution, it requires that

$$\left.\frac{\partial U}{\partial \xi}\right|_{\xi\to\infty} = 0, \qquad \left.\frac{\partial V}{\partial \xi}\right|_{\xi\to\infty} = 0, \tag{27}$$

leading to $B_1 = 0$ and $B_2 = 0$. Using Eqs. (25) and (26), Eq. (24) can be reduced to (up to the third order)

$$\partial_{\zeta\zeta}\theta = D_1\theta + D_2\theta^2 + D_3\theta^3, \tag{28}$$

where

$$\begin{aligned} D_1 &= -\left(8C_2 + 2\tan^2\theta_0 + \frac{\cos^2\phi\tan^2\theta_0}{c^2 - \cos^2\phi - K_1\sin^2\phi} + \frac{\sin^2\phi\tan^2\theta_0}{c^2 - K_1\cos^2\phi - \sin^2\phi}\right)/\Gamma, \\ D_2 &= -\left(16C_3 + 3\tan\theta_0 + \frac{3\cos^2\phi\tan\theta_0/2}{c^2 - \cos^2\phi - K_1\sin^2\phi} + \frac{3\sin^2\phi\tan\theta_0/2}{c^2 - K_1\cos^2\phi - \sin^2\phi}\right)/\Gamma, \\ D_3 &= -\left[32C_4 + 1 - \frac{4\tan^2\theta_0}{3} + \frac{\cos^2\phi\left(1/2 - 2\tan^2\theta_0/3\right)}{c^2 - \cos^2\phi - K_1\sin^2\phi} + \frac{\sin^2\phi\left(1/2 - 2\tan^2\theta_0/3\right)}{c^2 - K_1\cos^2\phi - \sin^2\phi}\right]/\Gamma. \end{aligned} \tag{29}$$

with

$$\Gamma = \frac{c^2}{\beta^2} - \frac{K_1 - \tan^2\theta_0 - 4C_2}{4} + \frac{\tan^2\theta_0\cos^4\phi}{6c^2 - 6\cos^2\phi - 6K_1\sin^2\phi} + \frac{\tan^2\theta_0\sin^4\phi}{6c^2 - 6K_1\cos^2\phi - 6\sin^2\phi}. \tag{30}$$

Eq. (28) admits an analytical solution in the form of

$$\theta = \frac{1}{E_1 \pm E_2\cosh\left(\xi/W\right)}, \tag{31}$$

where

$$\begin{aligned} E_1 &= -\frac{D_2}{3D_1}, \\ E_2 &= \sqrt{\frac{D_2^2}{9D_1^2} - \frac{D_3}{2D_1}}, \\ W &= \frac{1}{\sqrt{D_1}}. \end{aligned} \tag{32}$$

The existence of the two solutions defined by Eq. (31) requires that

(i) The width W f a soliton is a real number, yielding

$$D_1 > 0 \tag{33}$$

(ii) E_2 is a real number, yielding

$$\frac{D_2^2}{9D_1^2} - \frac{D_3}{2D_1} > 0 \tag{34}$$

(iii) the denominator in Eq. (31) is different from zero,

$$E_1 + E_2\cosh\left(\xi/W\right) \neq 0. \tag{35}$$

These conditions can lead to $D_1 > 0$ and $D_3 < 0$. It is worth noting that Eqs. (28)-(32) can be reduced to the soliton solution for monostable systems as reported in [2] (i.e., with $C_2 = K_2$ and $C_3 = C_4 = 0$). For the parameters that are deliberately chosen to achieve a critical nucleus size of 2×2 squares ($\theta_0 \approx 25.5°$, $\theta_M = 43°$, $K_1 = 0.2$, $C_2 = 0.051$, $C_3 = -0.115$, $C_4 = -0.579$, $\beta = 3.0556$), we find that soliton solution cannot be obtained for impact amplitude $A_0 = A_c$. However, the corresponding pulse propagation observed in the numerical simulations still behaves qualitatively similar to those reported in [2] where soliton solution can be obtained (the simulation results are provided in the following section).

Supplementary Note 3: Numerical characterizations

Anisotropy of the 2D transition wave

Here, we document the numerical simulation of a phase transition triggered quasistatically in a system of 30×30 squares. Snapshots from the numerical simulations are displayed in Supplementary Figure 6 for normalized times $T = 0$, 40, 75, and 100. A critical nucleus is formed by applying rotation θ_{in} to the 2×2 squares at the center. After nucleation, a phase transition is initiated and propagates outward throughout the rest of the metamaterial in the form of a transition wave. Moreover, we observe that the transition propagates outward anisotropically: it travels faster along the x and y axes than along the diagonals, and the wave fronts propagate along the diagonals of the system (i.e., $\pm 45°$ with respect to the x axis). To further corroborate this observation, we extract and plot in Supplementary Figure 7**b** the spatial profiles at $T = 40$ for all three degrees of freedom (i.e., normalized displacements U and V, and angle θ) along the horizontal and diagonal directions, as indicated by the black and magenta dots in Supplementary Figure 7**a**, respectively. In Supplementary Figure 7**c** and 7**d**, we display the contour plots of the spatio-temporal data of the angles along the horizontal and diagonal directions, respectively. The transition wave propagates considerably faster along the horizontal direction.

Other critical nucleus sizes

Supplementary Figure 8 shows that two different critical nucleus sizes are numerically determined for the following two sets of parameters: ($K_1 = 0.2$, $K_2 = 0.0336$, $\beta = 3.0568$) and ($K_1 = 0.2$, $K_2 = 0.0428$, $\beta = 3.0593$). By definition, critical nucleus is the smallest cluster size from which the new phase starts to grow. In general, we can start with an initial guess of the critical nucleus size, and perform numerical simulation to determine whether it is the correct one (i.e., whether a phase transition can be observed numerically). If not, we can increase the size and repeat the numerical simulation until the critical size is determined. Following the above procedures, for the first set of parameters, we numerically determine the critical nucleus size as 6 squares with a rectangular shape as shown in Supplementary Figure 8**b**. For the second set of parameters, we numerically determine the critical nucleus size as 12 squares with a "+" shape as shown in Supplementary Figure 8**d**.

Head-on collision of two pulses triggered by tensile impulses

We show in Supplementary Figure 9 the simulation result for a head-on collision of two pulses with same (negative) rotation, which is obtained with two tensile impulses for $A_0 = 0.306$. Supplementary Figure 9**a** displays snapshots of the wavefield before collision at $T = 15$, during collision at $T = 28$, and after collision at $T = 35$. Supplementary Figure 8**b** gives a spatiotemporal plot of the angle of the squares extracted along the propagation direction, and Supplementary Figure 9**c** gives the total kinetic energy of the system as a function of time. In this case, nucleation does not occur and the energy exchange between the the two components (i.e., translational and rotational) of the kinetic energy is almost negligible. As indicated in Fig.1**b** in the main text, the energy barrier between Phase C and Phase L is very high and requires very large negative rotation (θ_L). During the collision and before the energy barrier is reached, the rotational kinetic energy gained from the energy exchange is quickly absorbed by the torsional stain energy. Therefore, we observe that the energy exchange does not increase appreciably and is almost negligible when considering two tensile impulses.

Effect of propagation distance on collision-induced nucleation

To explore the effect of propagation distance on collision-induced nucleation, we consider three circular systems with different diameters $D = 24$, 30, and 36 (note that $D = 30$ is the reference case studied in the main text). In Supplementary Figure 10**a**-**f**, we report the snapshots of the wavefields and the energy of the nucleus highlighted in maroon for $D = 24$, 30, and 36. Based on the full-scale simulations, we numerically identify the critical energy barrier E_c^{nu}, the critical impact amplitude A_c, and the critical total input energy E_c^{in} for the three cases, which are reported in Supplementary Figure 10**g**. As expected, the critical impact amplitude A_c and total input energy E_c^{in} increase as the diameter increases, because the nonlinear pulse spreads in the 2D domain, and therefore its amplitude spatially decays as it propagates through the media. In contrast, the critical energy barrier E_c^{nu} shows no statistically significant change (the small differences may be caused by inevitable numerical errors).

To further investigate the spreading of the pulses mentioned above, we consider the propagation of a single pulse. Supplementary Figure 11**a** shows snapshots from the numerical simulation of a single pulse propagation at normalized times $T = 13.9$, 20.8, 27.8, and 34.7, and the corresponding spatial profiles of the pulse along

its propagation direction are given in Supplementary Figure 11**b**. We observe dispersion, especially in the direction perpendicular to propagation, which is qualitatively similar to the expected 2D dispersion behavior observed previously [2]. As a result, the amplitude of the pulse decreases as it propagates through the media.

Characterization of the anisotropic behavior of the nonlinear pulses

Similar to the previous discussion of transition waves, we show in Supplementary Figure 12 the contour plots for mode-I and mode-II pulses using the spatiotemporal data of the angles for two different impact amplitudes ($A_0 = 0.1$ and 0.3). From these contour plots, we can approximately calculate the wave speed for each case, as reported in Supplementary Figure 12. The wave speed of mode I is much faster than that of mode II. Moreover, the wave speeds associated with both modes slightly decrease as the impact amplitude increases from $A_0 = 0.1$ to 0.3. Moreover, we reported in Supplementary Figure 13 the snapshots for impact angle of $30°$. We find that the wave separate into two modes with different wave speeds. Comparing Supplementary Figure 13**a** and 13**b**, we observe that this separation behavior is more pronounced in a larger structure. These findings are consistent with previous work on a monostable system of rotating squares [2].

Control of the spatio-temporal shape of the nucleated phase

Here, we give an example to demonstrate how the spatio-temporal shape of the nucleated phase can be controlled by changing the impact angles. As shown in Fig. 4**b** in the main text and Supplementary Figure 14**a**, collision of two mode-I pulses triggered by impacts at the left and top boundary can lead to propagation of the nucleated phase along the diagonal, at $45°$ relative to the x and y axes. By moving the left impact to the right boundary, the new phase will grow predominantly along the other diagonal (i.e., $-45°$ relative to the x and y axes), as displayed in Supplementary Figure 14**b**.

Supplementary References

[1] B. Deng, J. R. Raney, V. Tournat, and K. Bertoldi. Elastic Vector Solitons in Soft Architected Materials. *Phys. Rev. Lett.*, 118(20):204102, 2017.

[2] B. Deng, C. Mo, V. Tournat, K. Bertoldi, and J. R. Raney. Focusing and mode separation of elastic vector solitons in a 2D soft mechanical metamaterial. *Phys. Rev. Lett.*, 123(2):24101, 2019.

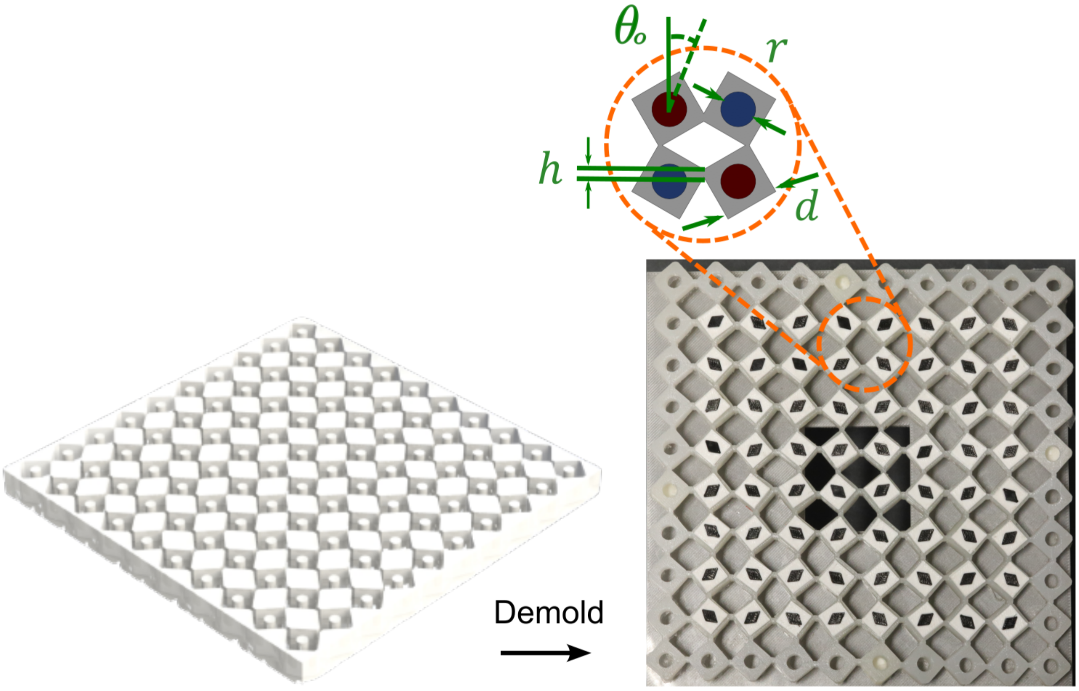

Supplementary Figure 1: Fabrication process of a system comprising 10 columns and 10 rows.

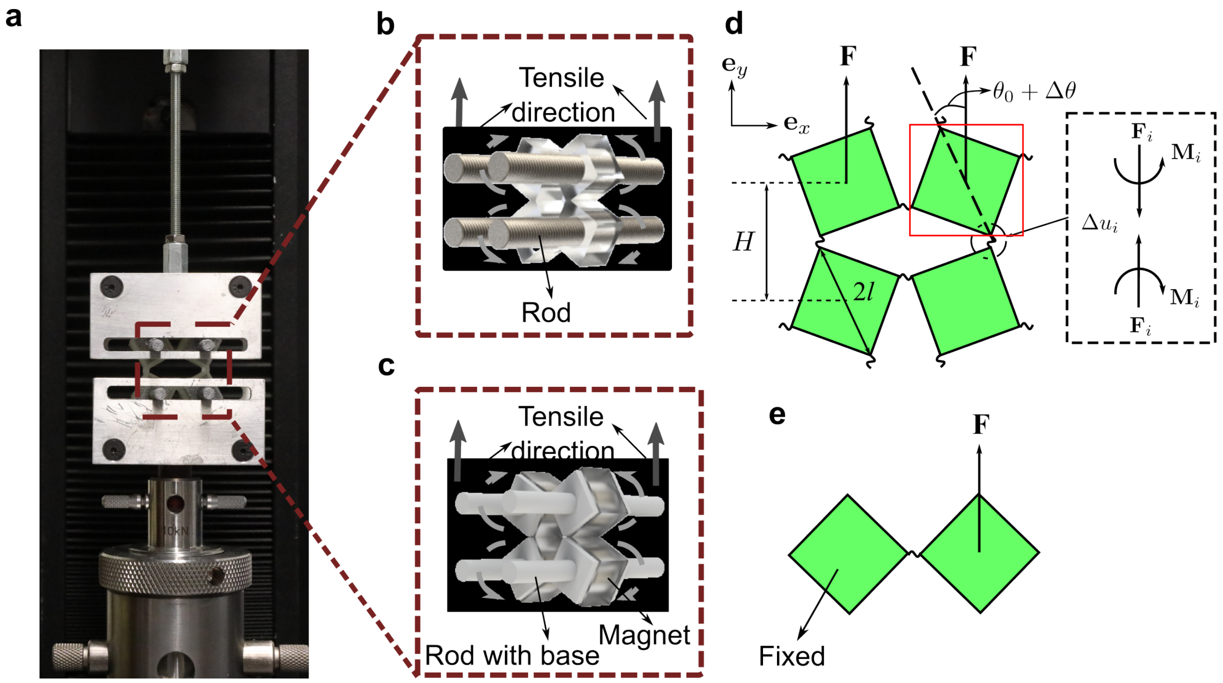

Supplementary Figure 2: **a** Tensile tests were conducted using an Instron model 68SC-5 equipped with a custom aluminum fixture. **b** Schematic of tensile test on a 2×2 unit without magnets (the rods are inserted through the holes of the squares). **c** Schematic of tensile test on a 2×2 unit with magnets (the rods with bases are attached to both sides of the squares). Black arrows indicate the direction of the applied force; grey arrows indicate the direction of rotation of each square. **d** Schematic of a 2×2 unit under tensile loading. **e** Schematic of a 1×2 unit under shear loading.

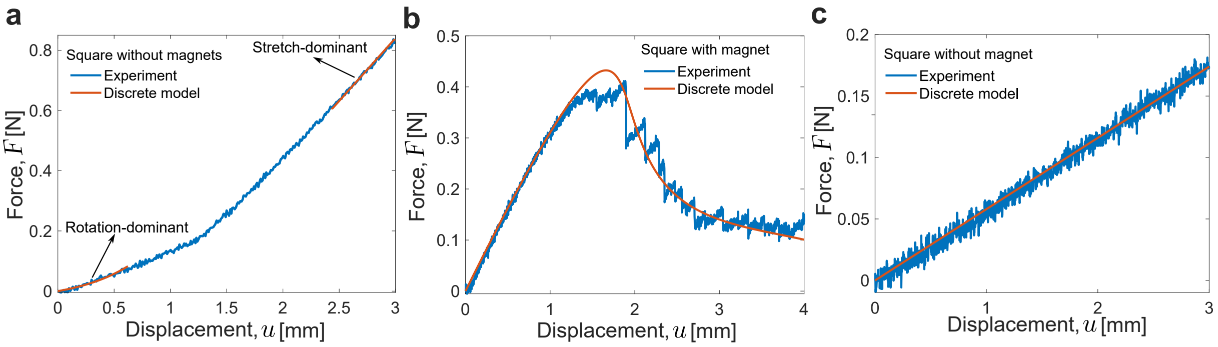

Supplementary Figure 3: Force-displacement relationships for a 2×2 unit under tensile loading **a** without magnets and **b** with magnets, and **c** a 1×2 unit under shear loading. Blue and red lines are obtained from experiments and discrete model, respectively.

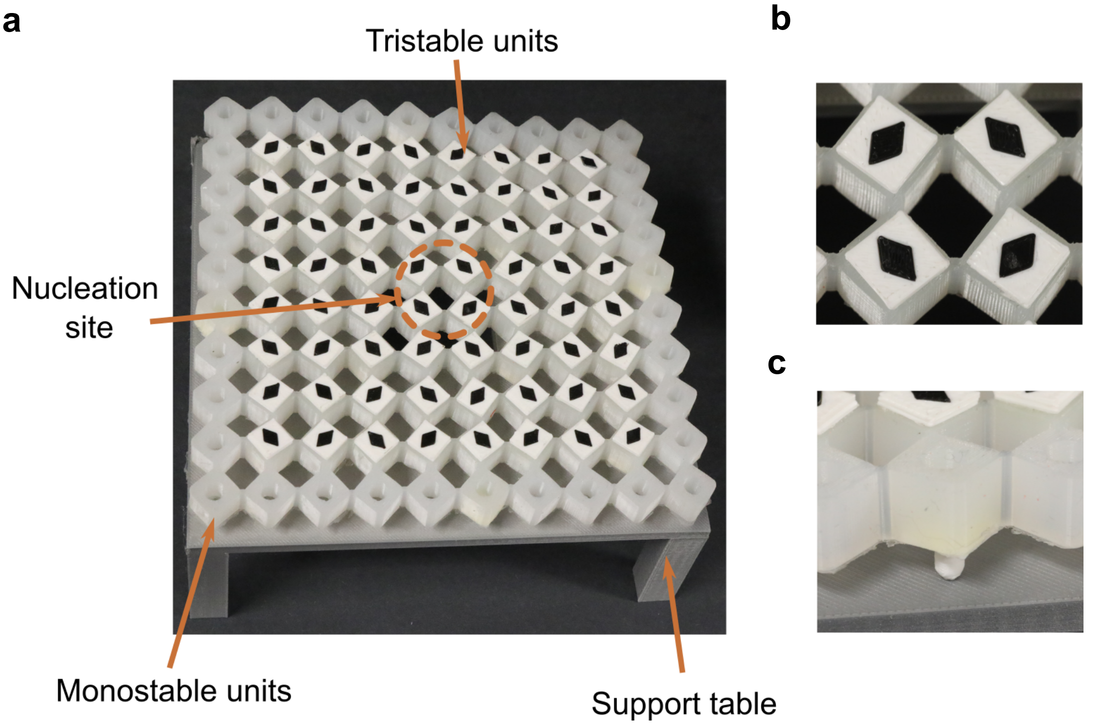

Supplementary Figure 4: **a** The experimental specimen rests on a support table. The orange circle indicates the nucleation site subjected to loading. **b** Top view of the center four squares, with diamond-shaped markers adhered to the center of each square. **c** Detailed view of a square with plastic feet (to reduce friction).

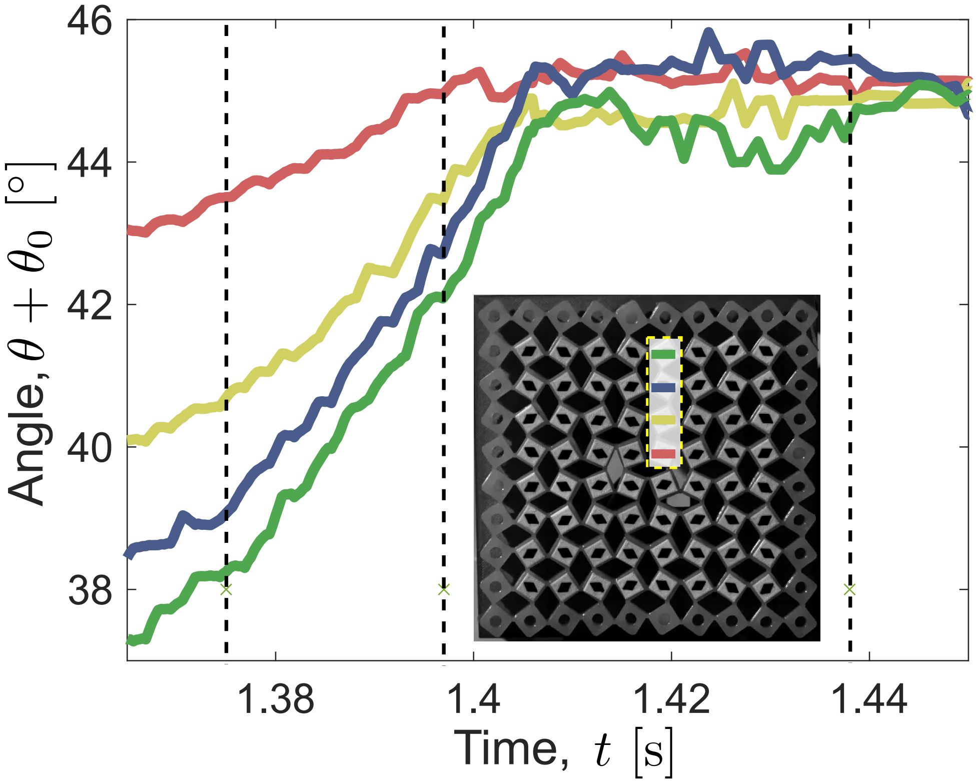

Supplementary Figure 5: Experimentally measured angles of the four squares indicated by the yellow dashed lines in the inset. The three vertical lines correspond to the times of the later three optical images displayed in Fig.1**c** in the main text.

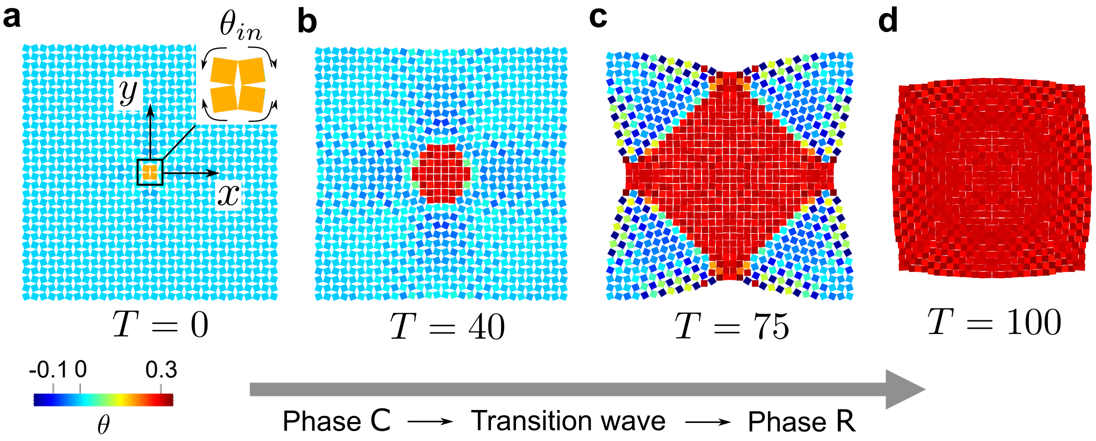

Supplementary Figure 6: Snapshots of quasistatic nucleation and growth observed via numerical simulation for a system comprising 30×30 squares at normalized times **a** $T = 0$, **b** $T = 40$, **c** $T = 75$, and **d** $T = 100$.

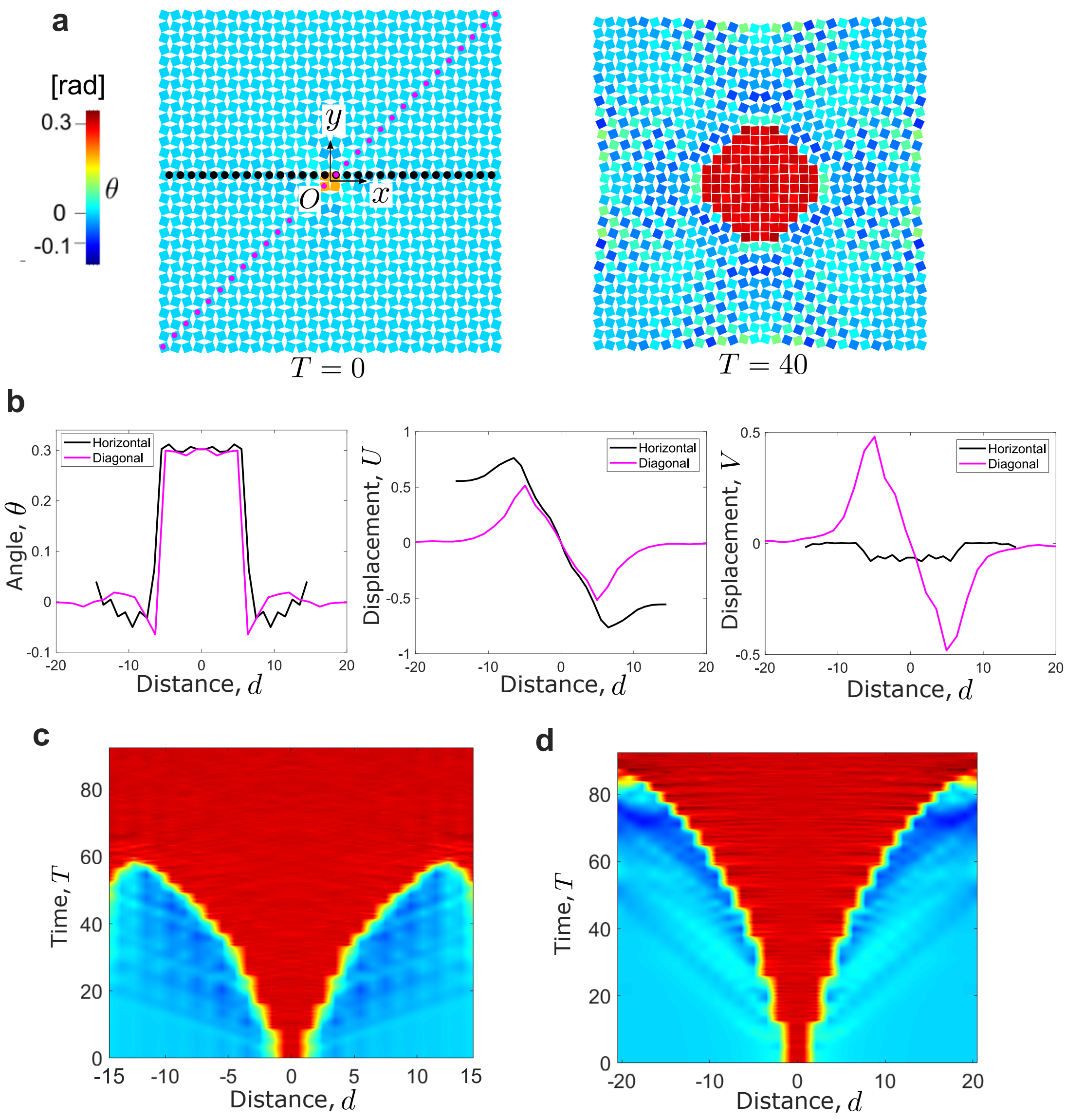

Supplementary Figure 7: Anisotropy of the transition wave. **a** Snapshots of the wavefield at $T = 0$ (T is set to 0 when the 2×2 squares at the center are rotated to Phase R) and 40. **b** Spatial profiles along the horizontal (black dots) and diagonal (magenta dots) directions for angle θ, displacement U, and displacement V. Contour plots of the spatio-temporal data for the angle along the **c** horizontal and **d** diagonal directions.

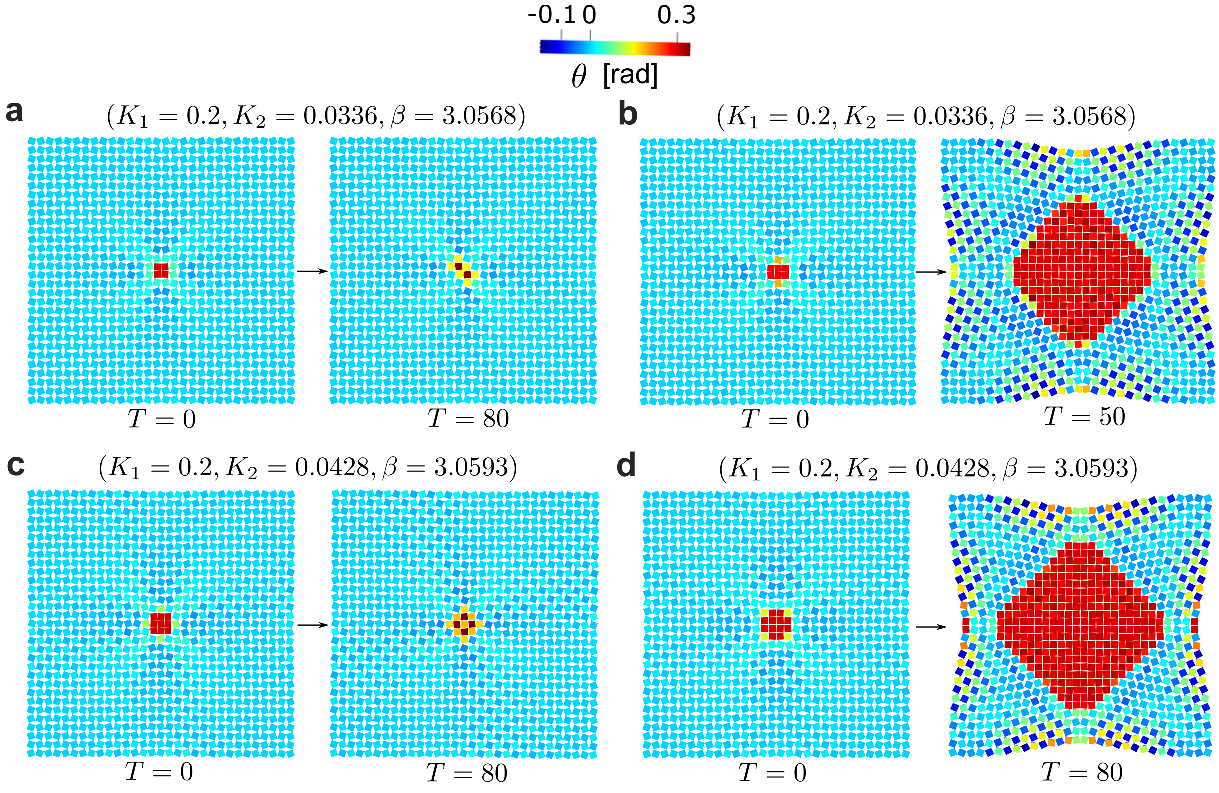

Supplementary Figure 8: Critical nucleus size for two sets of parameters. **a** 2×2 squares and **b** 6 squares with a rectangular shape rotated to Phase R initially for $(K_1 = 0.2, K_2 = 0.0336, \beta = 3.0568)$; **c** 3×3 squares and **d** 12 squares with a "+" shape rotated to Phase R initially for $(K_1 = 0.2, K_2 = 0.0428, \beta = 3.0593)$.

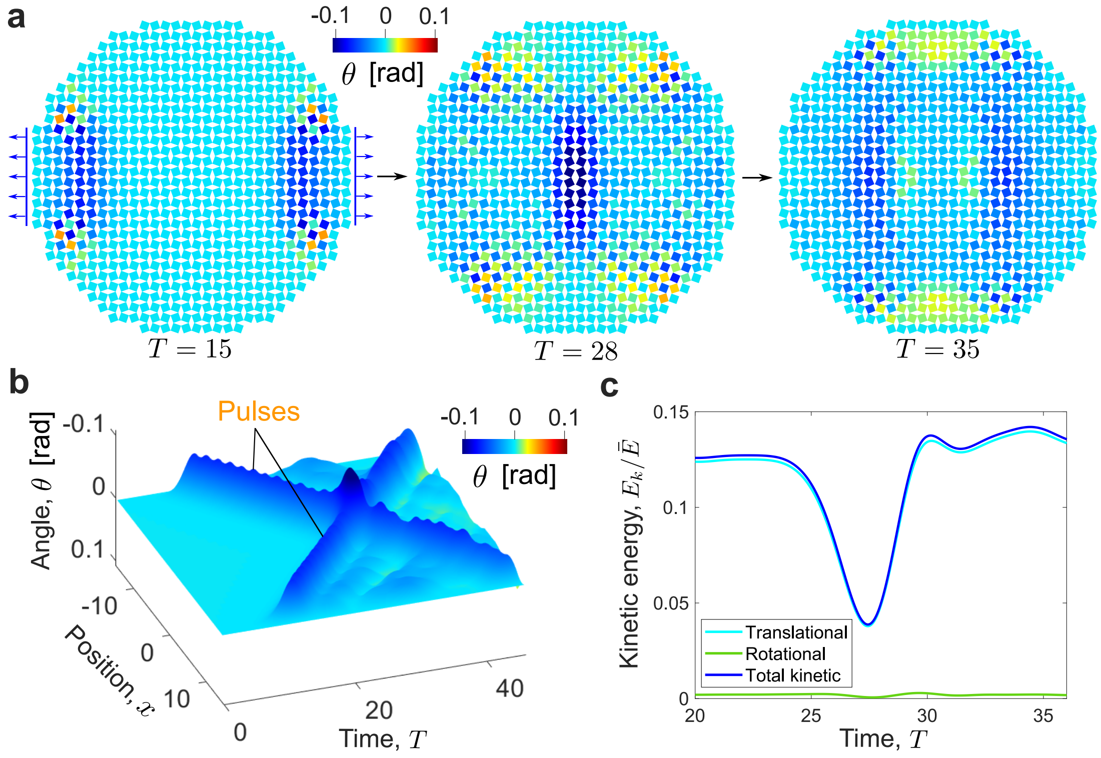

Supplementary Figure 9: Head-on collision of two pulses triggered by tensile impulses. **a** Snapshots of the wavefield before collision at $T = 15$, during collision at $T = 28$, and after collision at $T = 35$. **b** Spatiotemporal plot obtained from the numerical simulation, showing the angle θ for squares along the propagation direction as a function of time. **c** Kinetic energy of the whole structure as a function of time.

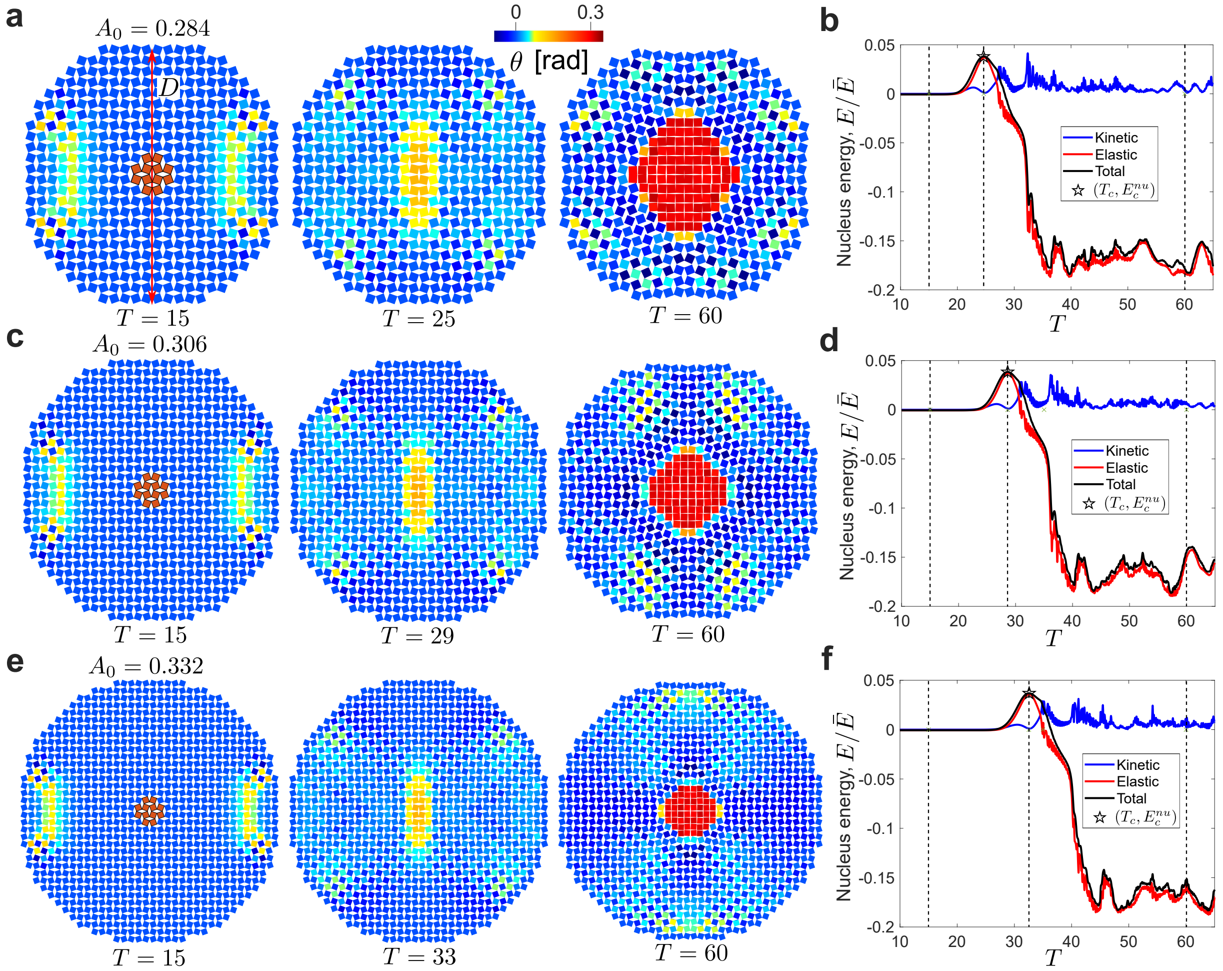

g

Diameter D	E_c^{nu}	A_c	E_c^{in}
24	3.81×10^{-2}	0.142	0.21
30	3.84×10^{-2}	0.153	0.2534
36	3.69×10^{-2}	0.166	0.2848

Supplementary Figure 10: Head-on collisions of nonlinear pulses in circular systems for three different diameters. Snapshots of wavefields and critical energy barrier of the nucleus highlighted in maroon for: **a** and **b** $D = 24$ and $A_0 = 0.142$; **c** and **d** $D = 30$ and $A_0 = 0.153$; **e** and **f** $D = 24$ and $A_0 = 0.142$. **g** Table: critical energy barrier E_c^{nu}, critical impact amplitude A_c, and critical total input energy E_c^{in} for $D = 24$, 30, and 36.

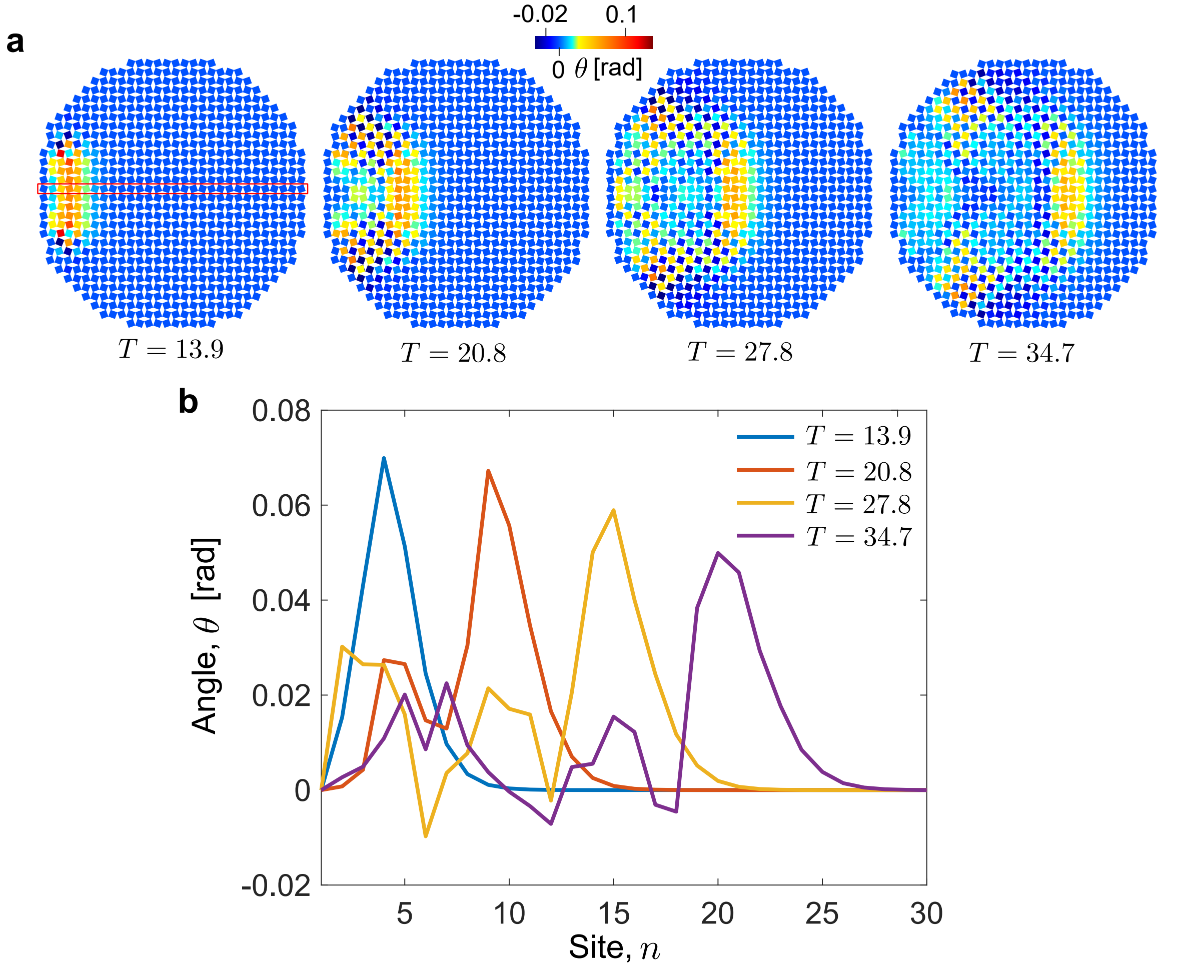

Supplementary Figure 11: Spreading of a single pulse. **a** Snapshots of wavefields for a single pulse at $T = 13.9, 20.8, 27.8$, and 34.7. **b** Spatial profiles of the pulse along the propagation direction highlighted by the red lines.

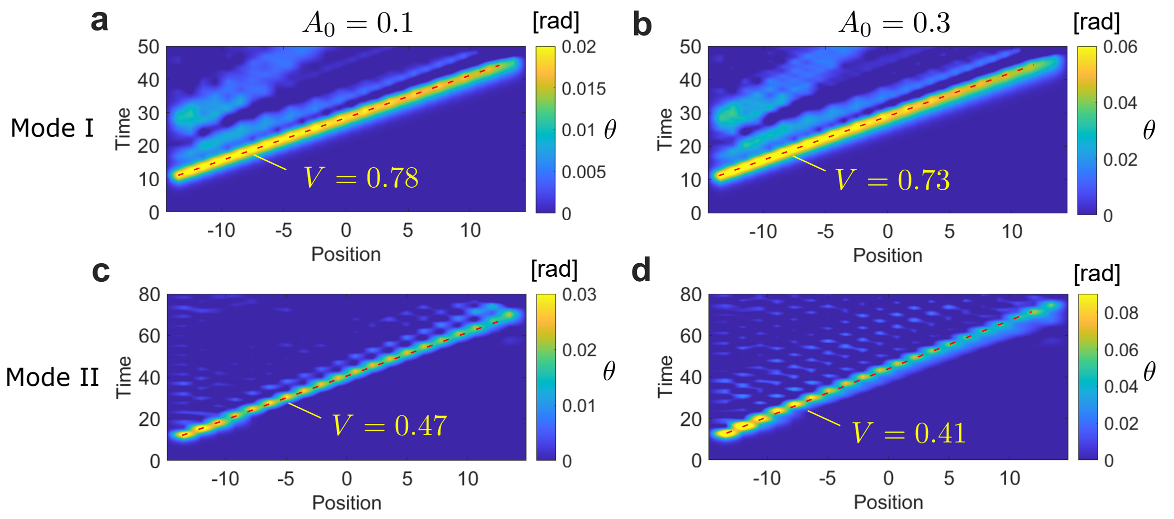

Supplementary Figure 12: Spatio-temporal plots extracted along the propagation direction for **a** pulse mode I with $A_0 = 0.1$; **b** pulse mode I with $A_0 = 0.3$; **c** pulse mode II with $A_0 = 0.1$; **d** pulse mode II with $A_0 = 0.3$.

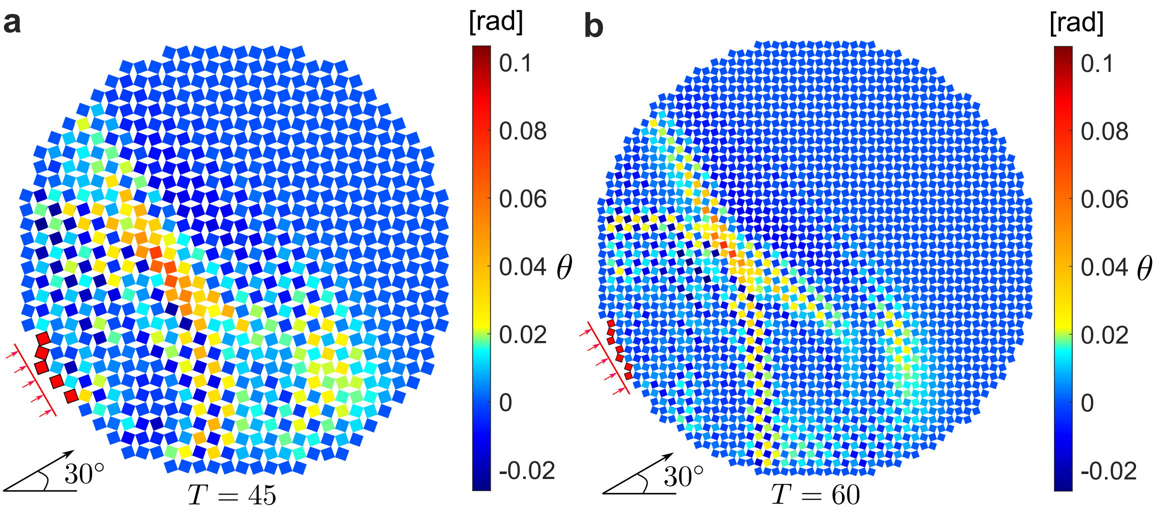

Supplementary Figure 13: Snapshots of wavefields with impact angle of 30° for **a** a circular system with 30 squares in diameter at $T = 45$ and **b** a circular system with 50 squares in diameter at $T = 60$. The red squares in **a** and **b** are those to which the impact is applied.

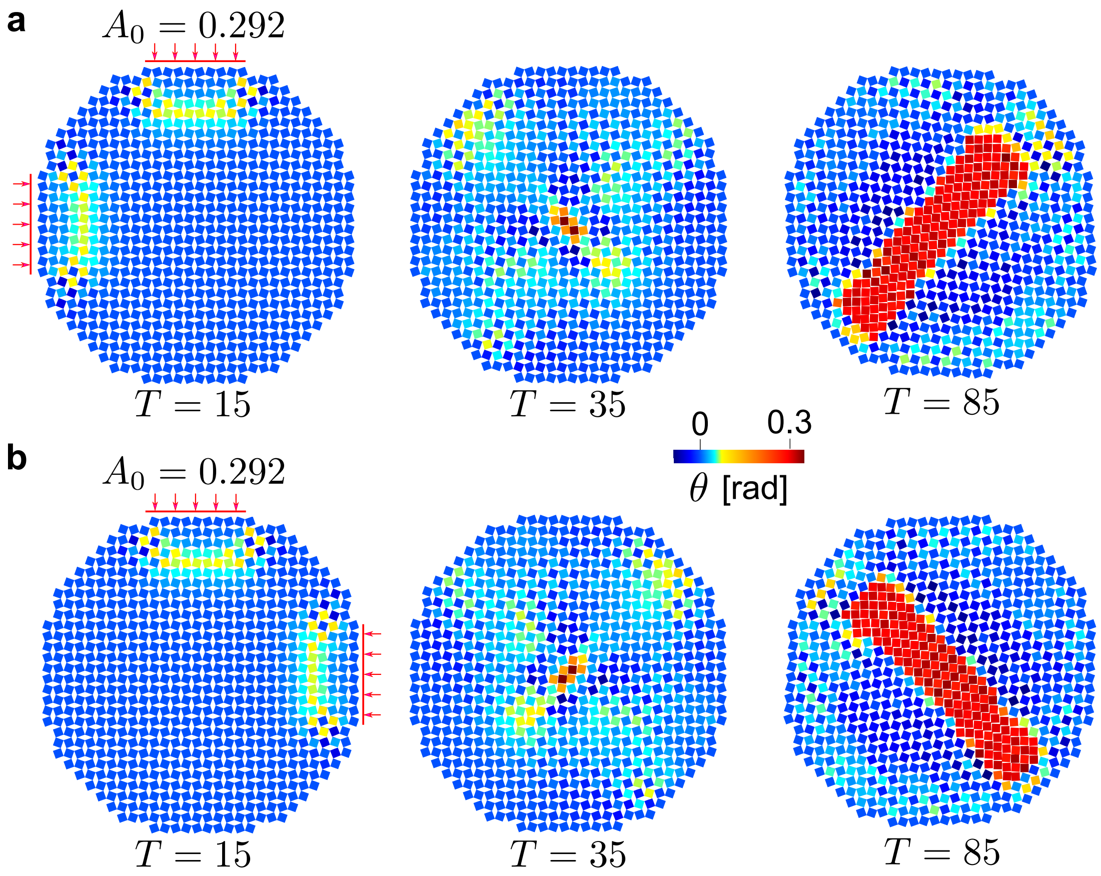

Supplementary Figure 14: Control of the spatio-temporal shape of the nucleated phase. **a** Collision of two mode-I pulses triggered by impacts at the left and top boundary with $A_0 = 0.292$, leading to propagation of the nucleated phase along the diagonal, at 45° relative to the x and y axes. **b** Collision of two mode-I pulses triggered by impacts at the right and top boundary with $A_0 = 0.292$, leading to propagation of the nucleated phase along the other diagonal, at -45° relative to the x and y axes.
