# Supplementary material for: Phase transitions in 2D multistable mechanical metamaterials via collisions of soliton-like pulses
Source: Nat Commun. 2024 Jan 6;15:333. doi: 10.1038/s41467-023-44293-w (PMC10771479; doi:10.1038/s41467-023-44293-w)
Supplement: Supplementary file 3 — Description of Additional Supplementary Files [file 41467_2023_44293_MOESM3_ESM.pdf]

Description of Additional Supplementary Files

File Name: Supplementary Movie 1
Description: Experimental observation of a phase transition in a 2D multistable metamaterial consisting of 10x10 rotating squares. A quasistatic load is applied at the center of the structure to trigger the phase transition. The phase transition propagates outward throughout the rest of the structure in the form of a transition wave, transforming it from its initial open state (Phase C) to a closed state (Phase R).

File Name: Supplementary Movie 2
Description: Numerical simulation of a phase transition induced quasistatically. Quasistatic loading is applied at the center of a structure comprising 30x30 squares, inducing the formation of a 2x2 critical nucleus and the propagation of a phase transition.

File Name: Supplementary Movie 3
Description: Nucleation of a phase transition via a head-on collision of soliton-like pulses. Two pulses with the same (positive) rotational direction are triggered by two compressive impulses of amplitude $A_0=0.306\equiv A_c$ at the left and right boundary of a circular-shaped system. When the two pulses collide at the center, a critical nucleus of 2x2 squares of Phase R is formed. Then, the new phase propagates outward via transition waves.

File Name: Supplementary Movie 4
Description: A head-on collision of two pulses with the same (positive) rotational direction for impact amplitude $A_0=0.3<A_c$. The critical nucleus is not formed and no phase transition is observed.

File Name: Supplementary Movie 5
Description: Control of the location of nucleation via the timing of the impulses. The simulation on the left is obtained with impulses initiated at $\Delta T=10$, while the simulation on the right is obtained with impulses initiated at $\Delta T=20$. ΔT denotes the time delay of the impact applied at the left boundary with respect to the other impact.

File Name: Supplementary Movie 6
Description: A head-on collision of two pulses with opposite rotational directions, with impact amplitude $A_0=A_c$. The two pulses pass through each other without nucleating a transition.

File Name: Supplementary Movie 7
Description: Effects of propagation direction on the ability of colliding pulses to nucleate the new phase. Collision scenarios include (appearing in order): 1. Collision of two mode-I pulses

propagating along x and y axes, with A_0=0.292; 2. Head-on collision of two mode-II pulses along the diagonal, with A_0=0.278; 3. Collision of two mode-II pulses propagating perpendicularly, with A_0=0.24; 4. Collision of a mode-I pulse and a mode-II pulse propagating along directions oriented 135 degrees with respect to one another, with A_0=0.302; 5. Collision of a mode-I pulse and a mode-II pulse propagating along directions oriented 45 degrees with respect to one another, with A_0=0.314.
